# Modeling and Simulation of Aggregation of Membrane Protein LAT with Molecular Variability in the Number of Binding Sites for Cytosolic Grb2-SOS1-Grb2

**DOI:** 10.1371/journal.pone.0028758

**Published:** 2012-03-01

**Authors:** Ambarish Nag, Michael Monine, Alan S. Perelson, Byron Goldstein

**Affiliations:** 1 Theoretical Biololgy and Biophysics Group, Theoretical Division, Los Alamos National Laboratory, Los Alamos, New Mexico, United States of America; 2 Theoretical Biololgy and Biophysics Group, Theoretical Division and Center for Nonlinear Studies, Los Alamos National Laboratory, Los Alamos, New Mexico, United States of America; German Cancer Research Center, Germany

## Abstract

The linker for activation of T cells (LAT), the linker for activation of B cells (LAB), and the linker for activation of X cells (LAX) form a family of transmembrane adaptor proteins widely expressed in lymphocytes. These scaffolding proteins have multiple binding motifs that, when phosphorylated, bind the SH2 domain of the cytosolic adaptor Grb2. Thus, the valence of LAT, LAB and LAX for Grb2 is variable, depending on the strength of receptor activation that initiates phosphorylation. During signaling, the LAT population will exhibit a time-varying distribution of Grb2 valences from zero to three. In the cytosol, Grb2 forms 1∶1 and 2∶1 complexes with the guanine nucleotide exchange factor SOS1. The 2∶1 complex can bridge two LAT molecules when each Grb2, through their SH2 domains, binds to a phosphorylated site on a separate LAT. In T cells and mast cells, after receptor engagement, receptor phosphoyrlation is rapidly followed by LAT phosphorylation and aggregation. In mast cells, aggregates containing more than one hundred LAT molecules have been detected. Previously we considered a homogeneous population of trivalent LAT molecules and showed that for a range of Grb2, SOS1 and LAT concentrations, an equilibrium theory for LAT aggregation predicts the formation of a gel-like phase comprising a very large aggregate (superaggregate). We now extend this theory to investigate the effects of a distribution of Grb2 valence in the LAT population on the formation of LAT aggregates and superaggregate and use stochastic simulations to calculate the fraction of the total LAT population in the superaggregate.

## Introduction

A ubiquitous method of initiating intracellular signals is through ligand-induced receptor aggregation [1]–[3]. However the role of aggregation in cell signaling is not restricted to bringing together the cytoplasmic domains of receptors, but occurs in multiple ways among multiple signaling molecules during the course of signaling. Scaffolding and adaptor proteins play important roles in cellular signal transduction [4]–[7]. The aggregation of scaffolding proteins differs from the aggregation of receptors in a fundamental way. The valence of a scaffolding protein for the species that induces its aggregation depends on its state of phosphorylation. The cytoplasmic domains of scaffolding proteins often have multiple binding motifs that, when phosphorylated, bind one or more types of signaling molecules. The number of phosphorylated binding motifs in a scaffolding protein molecule determines the valency of the molecule for other signaling molecules. Cross-linking of bivalent scaffolding molecules by other bivalent signaling molecules can result in the formation of chain-like aggregates. Cross-linking of multivalent scaffolding molecules by bivalent/multivalent signaling molecules can yield branched aggregates.

In the current article, we focus on the aggregation of the linker for the activation of T cells (LAT), a scaffolding protein that is essential for full mast cell and T cell function [8]. LAT is a membrane-localized adaptor protein which is primarily concentrated in microdomains by palmitoylation [5], [9]. It acts as a major signaling hub in the signaling network initiated by the aggregation of Fc
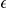
RI on mast cells and the aggregation of T cell receptors (TCRs) on T cells. LAT is a single chain protein composed of a transmembrane domain, a two amino acid extracellular domain, and a cytoplasmic domain containing nine tyrosine residues conserved in mice, rats and humans [10], [11]. The distal four LAT tyrosines play an essential role for both T cell [12] and mast cell function [13]. These four tyrosines, when phosphorylated, allow the SH2 containing phospholipase C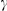 (PLC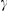) and the adaptor proteins Grb2 and Gads to associate with LAT [12], [14]–[16]. These adaptors [6], [17], [18] in turn recruit SOS1 and SLP-76 to LAT.

The distal three tyrosines on human LAT, Tyr171, Tyr191 and Tyr226, are located in YXNX sequences that bind the SH2 domain of Grb2 when the tyrosine in the motif is phosphorylated [19]. Hence, the valence of LAT for Grb2 can vary from zero to three depending on the state of LAT phosphorylation and in either mast cells or T cells that have been activated, there will be heterogeneity in the LAT population with respect to the number of Grb2 binding sites. In mast cells, the distribution of the LAT population over the different Grb2 valence states depends on the concentration of activated Syk molecules [20], [21], the kinase responsible for phosphorylating LAT, which in turn depends on the concentration of the extracellular allergen that cross-links IgE-Fc
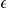
RI complexes. Similarly, In T cells the LAT valence distribution depends on the concentration of activated ZAP-70 molecules [22], which in turn depends on the extent of TCR activation.

The cytosolic adaptor molecule Grb2 consists of one SH2 domain flanked on each side by a Src homology 3 (SH3) domain [23], [24]. Both the SH3 domains of a Grb2 molecule can bind to proline-rich regions on a SOS1 molecule to form a 1∶1 Grb2-SOS1 complex. Binding of a second Grb2 involving both its SH3 domains to this complex can yield a Grb2-SOS1-Grb2 dimer capable of bridging two LAT molecules [6], each of which has at least one phosphorylated distal tyrosine. Although SOS1 has at least four binding sites for the SH3 domains of Grb2 [25], complexes of SOS1 and Grb2 with more than two Grb2 have not been reported. The interaction of this bivalent ligand (Grb2-SOS1-Grb2) with a trivalent scaffolding protein (fully phosphorylated LAT) offers the possibility of large aggregate formation since cross-linking of a trivalent species by a bivalent ligand can yield highly branched aggregates. Large clusters of LAT have been observed to form in T cells following T cell engagement [6], [26] and in mast cells following the aggregation of IgE-Fc
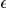
RI complexes [27], [28]. Experiments have shown that the cytosolic 2∶1 Grb2-SOS1 complex acts as a cross-linking agent between LAT molecules, leading to LAT cluster formation in T cells [6], [12], [29].

Previously, we presented an equilibrium model of LAT oligomerization for homogeneous populations of phosphorylated LAT, being content to compare the oligomerization properties of bi- and trivalent LAT in the presence of Grb2 and SOS1 [30]. The model predicted that over a certain range of concentrations of trivalent LAT, Grb2 and SOS1, a superaggregate of LAT formed in equilibrium with a distribution of smaller LAT aggregates and LAT monomers, i.e., there existed a sol-gel coexistence region [30]–[32]. We used the model to map the boundaries of the sol-gel region and predict the conditions under which a superaggregate can form, and the fraction of LAT molecules involved in the superaggregate for a given set of concentrations. For concentrations outside this range, a superaggregate (the gel phase) was predicted not to form. As has been shown in general for a bivalent ligand interacting with a bivalent receptor [31], for bivalent LAT there was no concentration range for which a sol-gel coexistence region existed. We now undertake a generalization of our previous equilibrium model to account for the distribution of LAT molecules over different valence states. Our focus is to understand how the presence of mono- and bivalent LAT alters the range of concentrations for which a gel phase can exist. Monovalent LAT will block LAT aggregation while bivalent LAT will prevent branching of LAT aggregates so we expect the presence of bi- and monovalent LAT to significantly reduce the range of concentrations over which a superaggregate can form. The main question that we address in the current article is how sensitive is the superaggregate formation to the presence of lower valence LAT. From the current article, it is evident that we can still expect to observe such aggregate formation for a range of physically relevant parameters. We have also calculated the fraction of the total LAT population in a superaggregate from stochastic simulations of the time evolution of the system. The simulation algorithm is an adaptation of a rule-based kinetic Monte Carlo approach discussed in detail in our previous work [30].

A related but different problem is to obtain an equilibrium model for the aggregation of bivalent receptors on cell surfaces, by a mixture of monovalent, bivalent and trivalent ligand species. Goldstein and Perelson (1984) developed the equilibrium theory for the clustering of bivalent cell surface receptors by trivalent ligands. We extend the theory in Goldstein and Perelson (1984) to include monovalent and bivalent ligand species in the Appendix S3 of this paper.

## Methods

### Theory

We present an equilibrium theory to calculate the LAT aggregate size distribution when there is differential phosphorylation of the LAT population resulting in heterogeneity in the number of Grb2 binding sites on LAT. We also present agent based simulations to check our equilibrium model [32]. For these simulations we use the rate constants as well as equilibrium constants that characterize the interactions in the model. The parameter values we use in our model are the same as in Nag et al. (2009). How they were obtained are fully discussed in that reference.

#### Calculation of total partition function for all species containing LAT molecules with variable valency

We first introduce the notation we will use and summarize our previous model. The cytosolic volume and surface area of the cell are represented by 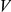 and 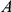 respectively. In contrast to our previous paper [30], where the entire LAT population is either bivalent or trivalent, the population of LAT in the current article is distributed between variable valence states for Grb2 - monovalent, bivalent and trivalent. The total cellular concentrations of monovalent, bivalent and trivalent LAT molecules are denoted by 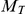, 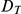 and 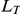 respectively so that the total numbers of membrane associated trivalent, bivalent and monovalent LAT per cell are 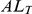, 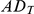, and 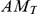 respectively. The total concentrations of Grb2 and SOS1 available to interact with LAT and each other are 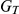 and 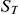 respectively, so that the total numbers of Grb2 and SOS1 in the cell are 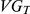 and 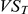 respectively. Grb2 contains an SH2 and two SH3 domains. It binds to phosphorylated LAT via its SH2 domain and to SOS1 through its SH3 domains. Grb2 can form both Grb2-SOS1 and Grb2-SOS1-Grb2 complexes [6], [33]. The equilibrium concentrations of free monovalent LAT, bivalent LAT, trivalent LAT, Grb2 and SOS1 are 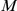, 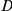, 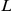, 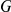, and 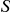, respectively.

**Figure 1:**
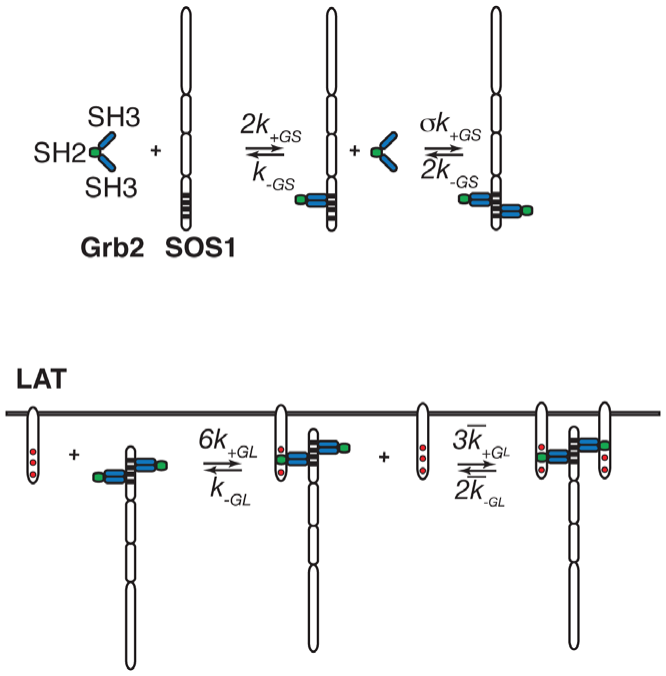
Reactions in the cytosol and just below the plasma membrane. a. The reactions involving the binding SH3 domains of Grb2 to the proline rich domains of SOS1 that result in the formation of a Grb2-SOS1-Grb2 complex. b. and c. Reactions involving the SH2 domain of Grb2 binding to phosphorylated LAT that result in the cross-linking of two trivalent LAT molecules by a Grb2-SOS1-Grb2 complex.

At equilibrium, the formation of the bivalent ligand Grb2-SOS1-Grb2 is characterized by an equilibrium constant 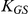 and a cooperativity factor 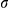. The equilibrium constant for the binding of the first Grb2 to a single Grb2 binding site on free SOS1 is 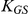. The forward and reverse rate constants for this binding event are 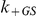 and 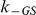, respectively. We take the binding of a second Grb2 molecule to a single Grb2 binding site in a 1∶1 Grb2-SOS1 complex to be negatively cooperative with the forward and reverse rate constants for the cross linking event as 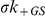 and 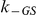. The corresponding equilibrium constant is 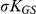. A 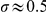 is consistent with the binding studies of Houtman et al. (2006). It follows from the definition of these equilibrium constants that 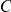, the equilibrium concentration of the bivalent ligand Grb2-SOS1-Grb2, is
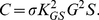
(1)The reactions leading to the formation of this complex are illustrated in Fig. 1a.

For simplicity we treat the three Grb2 binding sites on LAT as if they were identical and noncooperative and take the equilibrium constant for the binding between a LAT phosphotyrosine in a Grb2 binding motif and the SH2 domain of a free Grb2 molecule to be 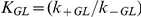. We also take 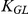 to be the same for Grb2, Grb2-SOS1 and Grb2-SOS1-Grb2 binding to a Grb2 binding site on LAT. (In this approximation we are neglecting the observed cooperativity that the binding of SOS1 to Grb2 or SOS1-Grb2 to Grb2 reduces the binding constant for the binding of Grb2 to LAT. For a discussion see Houtman et al. (2007)). We assume that the value of 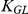 is independent of the valence state of the LAT molecule participating in Grb2 binding, i.e., 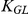 has the same value irrespective of whether it is a monovalent, bivalent or a trivalent LAT molecule that is binding free Grb2, or Grb2-SOS1, or Grb2-SOS1-Grb2.

As shown in Fig. 1b, the equilibrium cross-linking constant for binding to a phosphotyrosine on a second LAT molecule of the free Grb2 SH2 domain in a Grb2-SOS1-Grb2 species, already bound to a LAT molecule, is taken as 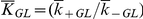. The value of 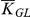 is assumed to be independent of the valency of the two LAT molecules being cross-linked.

Two LAT molecules can be cross-linked by a 2∶1 Grb2-SOS1 complex in another way. A 1∶1 complex of Grb2 and SOS1 can first bind to a LAT phosphotyrosine and this LAT-bound 1∶1 Grb2-SOS1 complex can then form a cross-link with a Grb2 molecule bound to another LAT phosphotyrosine. We denote the forward rate constant for this process by 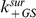, the reverse rate constant by 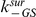 and the equilibrium constant by 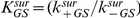. The principle of detailed balance demands that
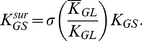
(2)Under the assumptions that 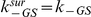 and 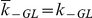,
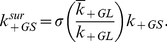
(3)


Since our theory is an equilibrium theory, which predicts the final equilibrium state of the system and not how this state is achieved by the system evolving in time, we do not need to explicitly consider these two different routes of LAT cross-linking in the theoretical derivations that follow. For the purpose of these derivations, we consider only the binding of the free SH2 domain in a LAT-bound Grb2-SOS1-Grb2 to a free phosphotyrosine on a second LAT molecule. On the other hand, for the stochastic simulations discussed later, which follow the evolution of the system in time, we have to explicitly consider the two different routes for LAT cross-linking.

In what follows, we assume that the dimer Grb2-SOS1-Grb2 cannot have its two Grb2 molecules bound simultaneously to the same LAT molecule. More importantly, we exclude all loop formation. If loops can form the number of free sites in an aggregate will be reduced, it will be more difficult to achieve superaggregate formation and the predicted size of the parameter space where a superaggregate can form will be over estimated. Very large aggregates of LAT have been detected in mast cells [27] which suggests that loop formation, if it occurs, is not a dominant effect.

We introduce the following non-dimensional parameters to develop the theory:
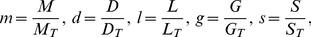
(4)

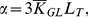
(5)

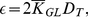
(6)

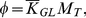
(7)

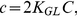
(8)

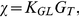
(9)

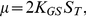
(10)

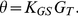
(11)

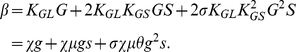
(12)

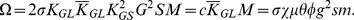
(13)


**Figure 2:**
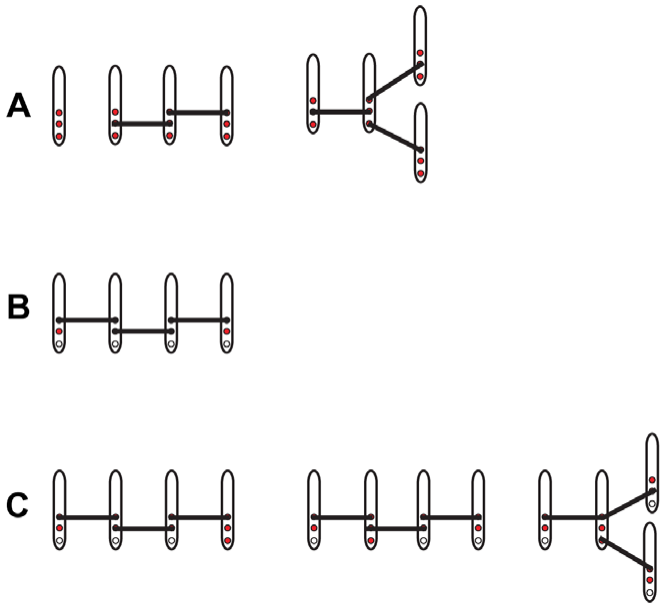
Some of the aggregates that contain four LATs and that only have bonds that participate in cross-linking. The line segment connecting two LAT molecules represents the bivalent ligand Grb2-SOS1-Grb2. (**A**) The total concentration of these two aggregates composed of four trivalent LAT contributes 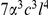 to the first term of 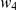. The first aggregate in part (A) contributes 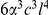 and the second aggregate contributes 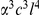 to the first term of 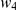. (**B**) The concentration of this aggregate of four bivalent LAT contributes 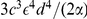 to the second term of 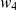. (**C**) The total concentration of these three aggregates composed of three bivalent and one trivalent LAT contributes 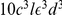 to the third term of 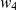. The first aggregate in part (C) contributes 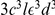, the second aggregate contributes 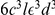 and the third aggregate contributes 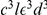 to the third term of 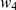.

In our previous paper [30], we derived for a model system comprising only trivalent LAT, Grb2 and SOS1, an expression for the dimensionless partition function 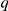, defined as the sum of the concentrations of all species containing trivalent LAT divided by the total trivalent LAT concentration. This partition function is given by
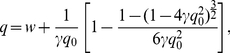
(14)where 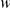, the concentration of species containing only one trivalent LAT molecule, can be written as 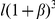, and 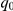 is the linear dimensionless partition function, i.e. it is the sum of concentrations of all possible linear chains of trivalent LAT molecules divided by the total trivalent LAT concentration. We have shown in our previous work [30], that 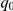 is given by
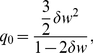
(15)where
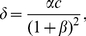
(16)and
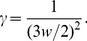
(17)In Appendix S1, we show that when the total LAT population is distributed over monovalent, bivalent and trivalent states in contrast to a homogeneous trivalent LAT population in Nag et al. (2009), the dimensionless partition function becomes
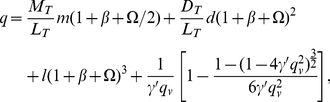
(18)where
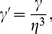
(19)

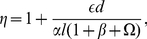
(20)

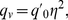
(21)

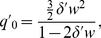
(22)and
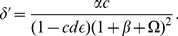
(23)The partition function 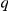 is defined as an infinite sum
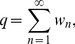
(24)where the surface concentration of species containing 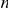 LAT molecules in all possible valence states, normalized by the total concentration of trivalent LAT is denoted as 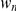. Expressions for 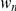 can be obtained by expanding the right hand side of Eq. (18) and summing the terms in that expansion that contain 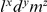 where 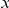, 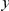 and 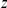 are integers with values greater than or equal to 0, such that 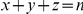. To illustrate, from Eq. (18) we obtained the following expressions for 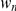 for LAT aggregates up to tetramers, i.e. 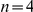, as:
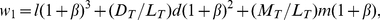
(25)

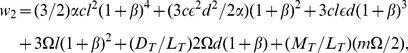
(26)

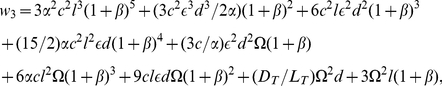
(27)and
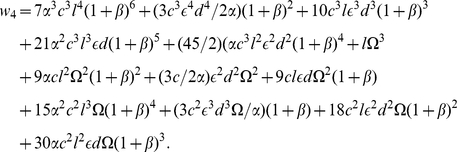
(28)The quantity 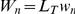 is the concentration of all aggregates that contain 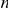 LAT molecules, irrespective of their Grb2 valences. This quantity is normalized by the total trivalent LAT concentration to obtain the dimensionless concentration 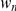, which is more tractable theoretically. Each separate term in the expression for a given 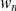 is the normalized concentration for all complexes having the same number of monovalent, bivalent and trivalent LAT. For example, the first term in the expression for 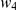, which is proportional to 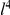, is the normalized concentration of all aggregates of four trivalent LATs. Of the twelve LAT binding sites in these aggregates, six are bound to Grb2-SOS1-Grb2 complexes that cross-linked two LATs while the remaining six are not involved in cross-linking. In general, if there are 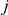 binding sites in an aggregate not involved in cross-links, the normalized concentration of the aggregate is proportional to 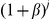. When 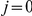 all sites on LAT are involved in cross-linking. Two such aggregates contribute to 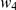, a trivalent LAT bound to three monovalent LAT and an aggregate of two bivalent and two monovalent LAT. Their normalized concentrations are the sixth and eighth terms in the expression for 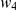. Shown in Figure 2 are the aggregates that contribute to the first three terms in 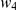 that have only bonds that cross-link two non-monovalent LATs.

Two quantities that will help us characterize the LAT aggregate size distribution are the average number of LATs per aggregate, 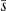, and the fraction of the total number of LAT in aggregates, 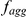, where
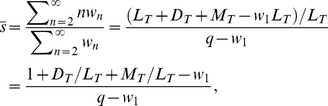
(29)and
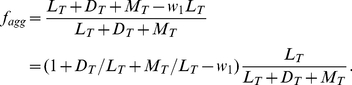
(30)


#### Size distribution of LAT aggregates

The reduced equilibrium concentrations, 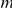, 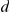, 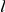, 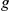 and 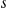, and hence the dimensional equilibrium concentrations, can be determined by solving the following conservation equations. These conservation equations are obtained from the partition function, Eq. (18), by noting that the partition function is a sum of normalized concentrations, each term of which is proportional to 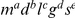, where the exponents are equal to the number of molecules of the given type in the aggregate, i.e, in an aggregate there would be 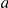 monovalent LAT, 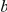 bivalent LAT, etc.. For a cell where LAT is distributed on a surface of area 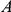, and the Grb2 and SOS1 that are not associated with LAT are distributed in a volume 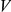, the conservation laws become
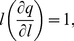
(31)

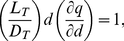
(32)

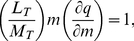
(33)

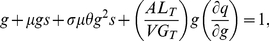
(34)and
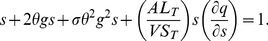
(35)Once 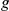, 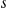, 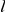 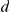 and 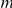 are determined by solving the above conservation equations, one can, for example, evaluate 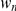 for 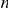 between 1 and 4 using Eqs. (25)–(28). The distribution of LAT aggregate sizes can be obtained by plotting 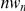 versus 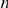.

#### Formation of a gel-like phase

Cross-linking of bivalent LAT molecules by a bivalent ligand results in only linear aggregates and cannot yield a gel like state [31]. It has been shown in Nag et al. (2009), that for trivalent LAT, where branched aggregates can form, the possibility arises that a gel-like state, i.e., a superaggregate, can form. In the course of aggregation of trivalent LAT, a point may be reached at which the probability of having an infinite sized aggregate changes from zero to a positive value [31]. This point is called the gel point. We currently investigate the conditions that the gel point should satisfy in the presence of multiple valence states of LAT.

We define 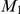 and 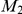 as the first and second moments respectively of the LAT aggregate distribution. Then,
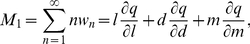
(36)and,
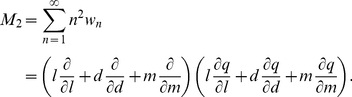
(37)The first moment is the fraction of trivalent LAT in finite-sized aggregates. In the absence of infinite aggregates 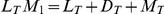 is the conservation law for LAT. The second moment is the average size of all entities containing trivalent LAT. At the gel point, 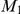 equals 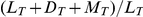 but 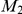 diverges indicating the presence of supperaggregates. From the validity of the conservation equations Eqs. (31)–(35) and the divergence of 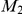 at the gel point, we show in Appendix S2 that at the gel point, the following equation is valid as well.
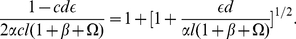
(38)


For given total numbers of Grb2, SOS1 and monovalent LAT molecules molecules in the cell, we can simultaneously solve Eqs. (31)–(35), (38) to obtain the two phase (sol-gel) region in the parameter space spanned by the number of bivalent LAT molecules and the number of trivalent LAT molecules in the cell.

Alternatively, for given total numbers of Grb2, monovalent and bivalent LAT molecules present in the cell, we can obtain the two phase region in the parameter space spanned by the number of SOS1 molecules and the number of trivalent LAT molecules in the cell.

### Stochastic Simulations

We have adapted a rule-based kinetic Monte Carlo (kMC) approach [32], [34] to numerically simulate LAT-GRB2-SOS1 interactions. The main advantage provided by the rule-based kMC over standard methods for simulation stochastic dynamics of biochemical systems [35], [36] is the avoidance of the combinatorial explosion of the number of possible species and reactions that arises from the interactions of multivalent molecules [37], [38]. Particularly, conventional chemical kinetics methods could not be used for dynamical simulation of LAT aggregation in the gel phase regime since the number of equations that would need to be integrated would be effectively infinite. An overview and a detailed description of the method is given in Nag et al. (2009).

The main assumption underlying the kMC model of Nag et al. (2009) is that the binding kinetics at free LAT sites does not depend on the occupancy of other sites on the same LAT molecule. This assumption allows us to condense the reaction network of all possible configurations to a much smaller network of reactants as no local constraints on binding to LAT need to be taken into account. Thus, each binding interaction involving LAT requires sampling only from an array of free LAT sites; complexes of multiple LAT molecules are included automatically. Note that the model of Nag et al. (2009) considers LAT molecules of valence three only. Here, to include variability in the valence of LAT, we modify the data structure of the kMC model of Nag et al. (2009). We create an array of 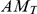 sites of monovalent LAT molecules, 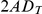 sites of bivalent LAT molecules and 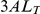 sites of trivalent LAT molecules. By definition, each LAT site is associated with a specific LAT molecule. Therefore, we also create an array of indexed LAT molecules, where molecule index 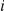 is used to determine the valence of LAT in accordance with the following rule: if 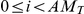, the 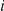
th molecule is monovalent; if 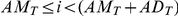, the 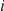
th molecule is bivalent; if 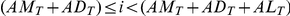, the 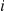
th molecule is trivalent. Simulation procedure of the modified kMC model follows the steps described in Nag et al. (2009).

We use the simulations to track the aggregate size distribution in time. As discussed earlier in the Theory section, we explicitly consider in the simulations, two different ways by which a cross-link can form between two LAT molecules, characterized by two different sets of rate constants - i) 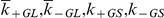 and ii) 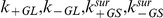. The final output is obtained by averaging over multiple runs. The system size is set to the total number of molecules, and it can also be rescaled by multiplying bimolecular rate constants by a volume factor, 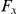, and dividing 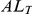, 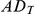, 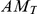, 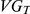 and 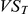 by 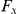.

## Results

In this section, we present results obtained from the equilibrium model and stochastic simulations. For convenience in representation, the terms 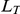, 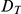, 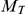, 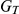 and 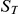 in the current section and in the related figures are used to refer to the total numbers per cell of trivalent LAT, bivalent LAT, monovalent LAT, Grb2 and SOS1 respectively and not to their concentrations as in the Methods section. The values of kinetic and other model parameters used in the equilibrium model calculations and in the stochastic simulations have been provided in Table 1.

**Table 1:** Parameters used in the simulations and the equilibrium model calculations.

Parameter	Value(s)
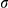	0.5
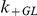	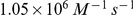
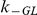	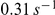
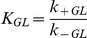	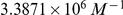
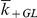	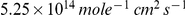
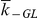	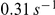
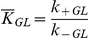	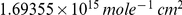
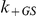	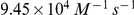
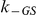	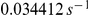
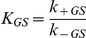	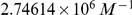
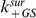	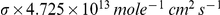
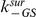	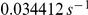
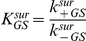	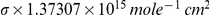
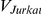	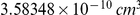
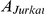	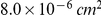

This table furnishes values of parameters used in the simulations and the equilibrium model calculations. 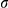 is a negative cooperativity factor for the binding of a second Grb2 to a Grb2-SOS1 complex (see Eq. (1)) [6]. The equilibrium constants for the binding of Grb2 to one of the three terminal phosphotyrosines on LAT range from 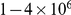 M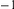 [6], [33], [45]. In our model the affinities for these three binding sites are identical. We take 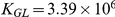 M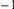. The values for 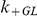 and 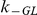 are from biacore studies of the binding of the SH2 domain in a Grb2-SOS1 complex to an eleven peptide sequence from the cytoplasmic domain of EGFR that includes the Grb2 binding site pY1068 [48]. The values of these rate constants have no effect on any of the equilibrium results. The value of the equilibrium crosslinking constant 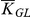 is estimated from the 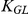 value as in Nag et al. (2009). The rate constant 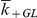 is calculated using the values of 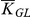 and 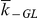 which we assume equals 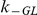. The value of 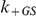 is taken from [49]. The dissociation constants for the binding of the Grb2 SH3 domain to the N-terminal and C-terminal proline-rich regions of SOS1 are 260 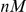 and 510 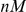 respectively [6]. In our model we do not distinguish between the two SH3 binding sites on SOS1 and take 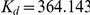 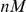, the geometric mean of the two values. The value of 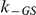 is calculated using the value of 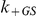 and the geometric mean of the 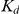 values. The value of 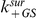 is estimated using Eq. (3) and taking 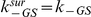. The diameter of the Jurkat cell, 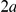, has been measured by Rosenbluth et al. [50] to be 11.5 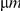 with the cytosol taking up about 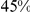 of the total cell volume. 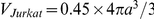 is the cytosolic volume. The surface area of the Jurkat cell is taken to be approximately twice the area of a sphere of diameter 11.5 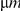. In the stochastic simulations, only unimolecular rate constants can be used directly so the solution bimolecular rate constants are scaled by the cytosolic volume 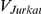 and the surface bimolecular rate constants are scaled by 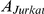. The exact values of the parameters we used are given in the table to allow our results to be reproduced. The accuracy of the parameters that have been determined by experiment is at best two significant figures.

### Size distribution of LAT aggregates

**Figure 3:**
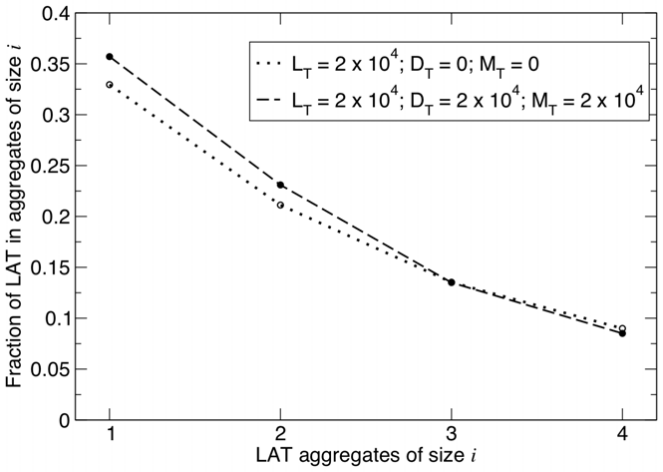
Comparison of size distribution of LAT aggregates for a homogeneous (open circles) and heterogeneous population of LAT (solid circles). For both homogeneous and heterogeneous LAT populations, the number of trivalent LAT is identical and equal to 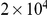 per cell, but for the heterogenous population of LAT also present are 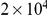 monovalent LAT and 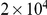 bivalent LAT. For both cases the numbers per cell of Grb2 and SOS1 were 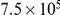 and 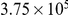 respectively. The simulation results were obtained from a single simulation run with 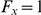. The theoretical results were obtained by solving Eqs. (31)–(35) simultaneously for 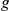,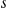,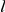,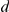 and 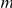 and then calculating 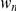 from Eqs. (25)–(28). To obtain the fraction of LAT present in an aggregate of a particular size, 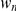 has to be multiplied by 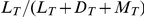 since 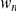 is obtained by dividing 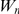 by 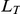. The open circles connected by dotted lines is for the homogeneous LAT population.

Shown in Fig. 3, for two different LAT populations, are the predicted LAT aggregate size distributions from simulation and from the equilibrium theory developed in the previous section. The predictions from simulation and theory for these values of Grb2, SOS1 and LAT are indistinguishable. For both curves the Grb2 and SOS1 populations were set equal to 7.5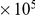 and 3.75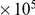 per cell, respectively, values typical for Jurkat T cells [30]. Unless stated, these are the values we will use in the examples discussed in this section. The solid curve corresponds to a heterogenous LAT population of 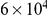 per cell, equally divided among monovalent, bivalent and trivalent LAT. The dotted curve corresponds, to a homogeneous population of 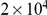 trivalent LAT so that in both cases the concentration of trivalent LAT is the same. From Eqs. (29) and (30) we calculate that for the heterogeneous population of LAT the average LAT aggregate size, 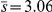 and the fraction of LAT in aggregates of size five or greater is 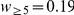. For the homogeneous population of trivalent LAT, 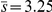 and 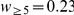. As we see in this example, the presence of monovalent and bivalent LAT (solid line) modestly increases the amount of LAT not in aggregates (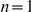 in Fig. 3) and decreases the amount of LAT in larger aggregates (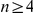).

### Formation of macromolecular aggregates

In this section, we explore the regions of concentration space that allow for the formation of a gel phase where super aggregates can form. In Nag et al. (2009), where we considered only homogeneous LAT populations, we were able to obtain an analytic expression for the fraction 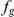 of LAT molecules in the gel phase. For a heterogeneous population of LAT the problem is more difficult and we have instead used stochastic simulations to calculate 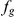 for specific values of the Grb2, SOS1 and LAT concentrations. In the simulations we define the number of LAT molecules in the gel phase as the number of LAT molecules of all three valencies in the largest aggregate averaged over time, once equilibrium has been established. To speed the simulations we reduced the system size, the volume and all the molecular populations, by a factor of 10 (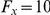). This scaling reduced the size of the superaggregate by a factor of ten compared to the full system, but did not affect the predicted fraction of LAT in the gel phase.

**Figure 4:**
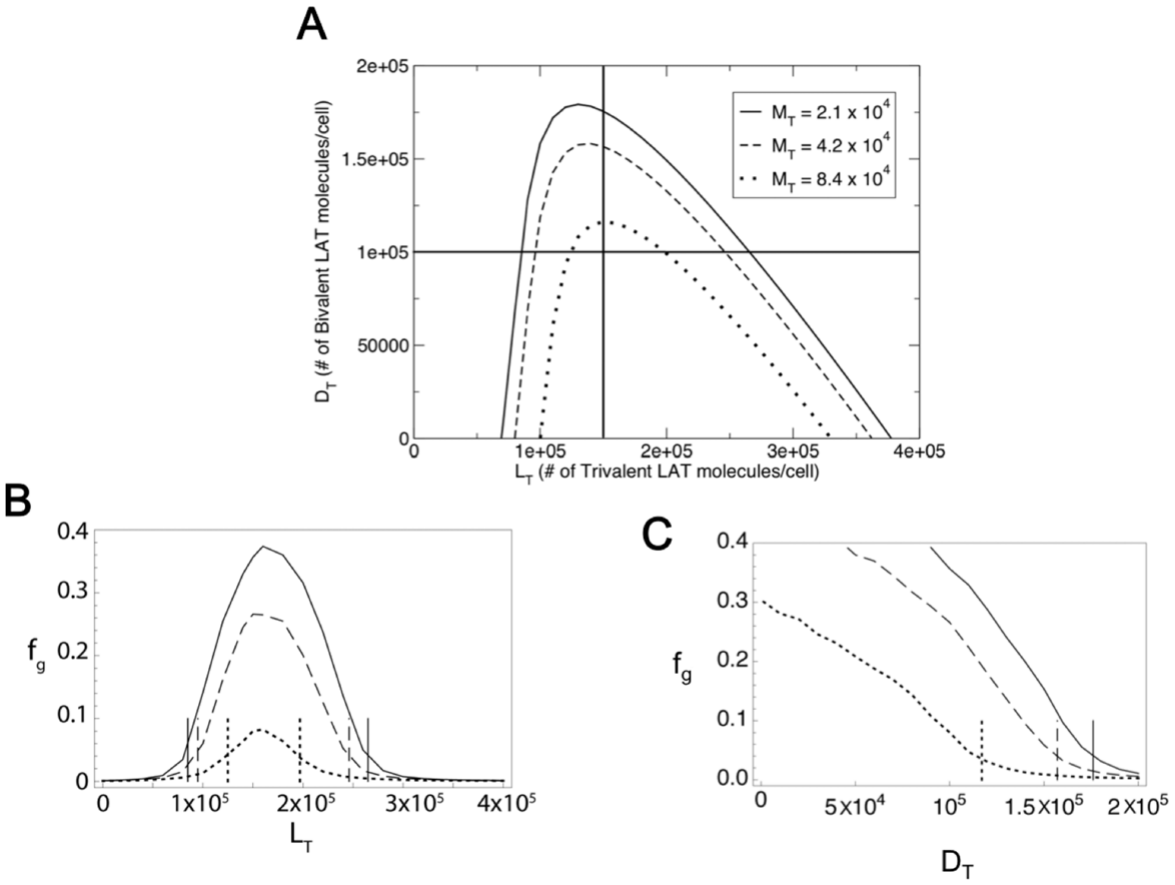
Two phase regions in space spanned by bivalent and trivalent LAT concentrations. (**A**) Two phase(sol-gel) regions for Grb2 and SOS1 cellular populations of 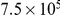 and 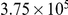 molecules per cell respectively. The population of monovalent LAT is fixed at three different values, 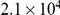 molecules per cell (solid line), 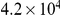 molecules per cell (dashed line) and 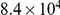 molecules per cell (dotted line). The solid horizontal and vertical lines correspond to the cellular populations of bivalent LAT (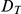) and trivalent LAT (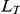) being fixed at 10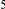 and 1.5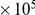 molecules per cell respectively. (**B**) Plot of 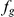, the fraction of the cellular inhomogeneous LAT population present in the gel phase, as a function of the cellular trivalent LAT population 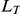, as predicted from stochastic simulations. The cellular bivalent LAT population (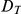) is fixed at 10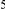 molecules per cell. In sync with (A), the solid, dashed and dotted lines correspond to fixed cellular monovalent LAT populations (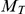) of 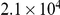, 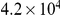 and 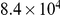 molecules per cell respectively, and cellular Grb2 and SOS1 populations are fixed at the same values as in (A). The vertical lines correspond to the theoretically predicted boundaries between the one phase (sol) and two-phase (sol-gel) regions obtained by varying 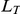 along the horizontal line in (a) corresponding to a fixed 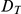 value of 10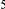 molecules per cell. (**C**) Plot of simulated 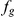 values as a function of 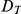 keeping 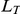 fixed at 1.5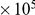 molecules per cell, and Grb2 and SOS1 fixed at the same values as in (A), for each of the three fixed monovalent LAT populations (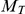) in (A). The vertical lines correspond to the theoretically predicted boundaries between the one phase (sol) and two-phase (sol-gel) regions obtained by varying 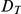 along the vertical line in (A) corresponding to a fixed 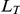 value of 1.5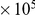 molecules per cell.

In Fig. 4a we consider three different total concentrations of monovalent LAT with 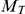 ranging from 2.1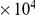 to 8.4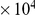 molecules/cell. Keeping 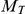 fixed at each concentration, we obtain from the equilibrium theory, Eqs. (31)–(35), (38), the boundary of the sol-gel region in the concentration space spanned by the total bivalent and trivalent LAT concentrations, 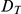 and 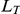. As expected, since monovalent LAT acts as a chain terminator, the higher the concentration of monovalent LAT, the smaller the sol-gel region in the 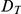-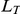 concentration space.

For each 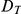 value in the two phase region, there exist two critical 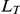 values which mark the onset of gel formation. Superaggregates cannot form below the lower value 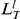 and above the upper value 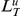. Below 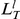, the total concentration of trivalent LAT relative to the total concentrations of Grb2, SOS1, bivalent and monovalent LAT is insufficient to yield a superaggregate. The larger is 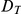, the greater is the number of trivalent LAT molecules necessary for the onset of the gel formation, so that 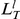 increases with 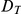. Above 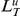, the number of trivalent LAT molecules is so high that there are not enough cross-linking Grb2-SOS-Grb2 species for superaggregate formation. This shortage is accentuated by the presence of bivalent and monovalent LAT competing with trivalent LAT for the cross-linking species, resulting in 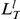 decreasing with 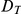.

In Fig. 4b, for each of the three 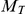 values in Fig. 4a, we have plotted 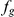 obtained from simulations as function of 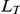. In the simulations, 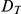 was fixed at 10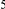 per cell and 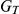 and 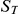 held at their usual values. The vertical lines correspond to the predicted boundaries from the equilibrium theory, i.e., the values of 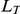 at which 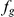 goes to zero. The 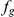 values obtained from the simulations have small but finite non-zero values at the predicted boundaries. This discrepancy arises because the equilibrium theory assumes the size of the system is infinite whereas the simulations are for a finite system. For a finite system there is no true transition from sol to sol plus gel and this is reflected in the way the simulated 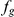 curves approach the x-axis. Since the simulations were done on a system whose volume was reduced by a factor of ten this discrepancy is accentuated.

In Fig. 4c, we plot the 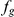 values as a function of 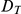, keeping 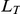 fixed at 1.5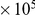 per cell and 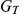, 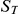 and 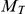 at the same values as in Fig. 4b. One phase boundary exists for such 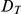 variation for each fixed 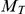 value. Again, the horizontal lines in Fig. 4c indicate the predicted boundaries from the equilibrium theory for the three fixed 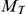 values in Fig. 4a. As in Fig. 4b, the simulated values for 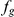 are small but not zero at these values, presumably due to finite size effects.

**Figure 5:**
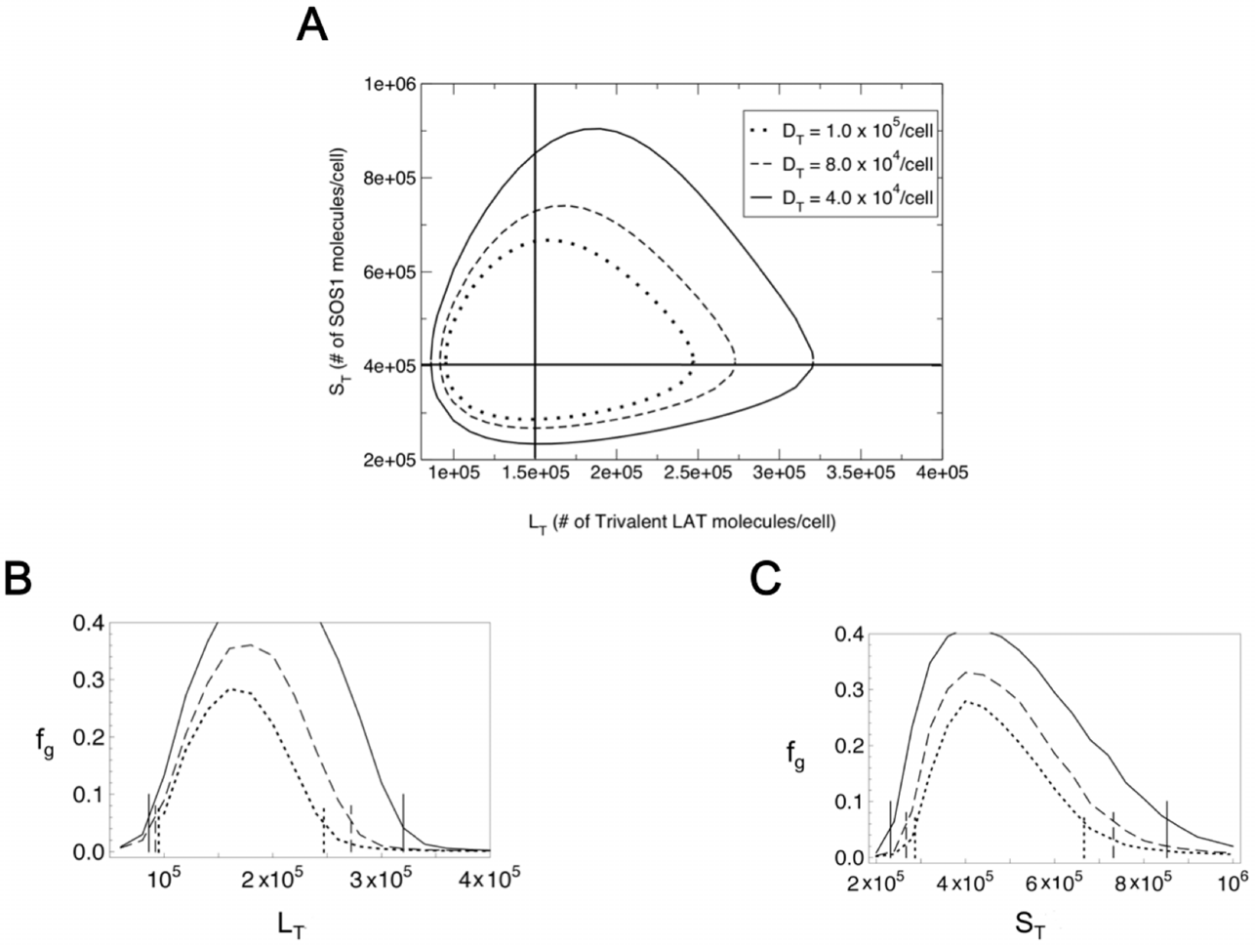
Two phase regions in space spanned by SOS1 and trivalent LAT concentrations. (**A**) Two phase(sol-gel) regions for Grb2 and monovalent LAT cellular populations of 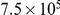 and 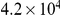 molecules per cell respectively. The population of bivalent LAT is fixed at three different values, 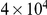 molecules per cell (solid line), 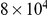 molecules per cell (dashed line) and 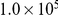 molecules per cell (dotted line). The solid horizontal and vertical lines correspond to the cellular populations of SOS1 (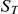) and trivalent LAT (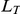) being fixed at 4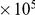 and 1.5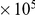 molecules per cell respectively. (**B**) Plot of 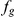, the fraction of the inhomogeneous LAT population present in the gel phase, as a function of the trivalent LAT population 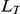, as predicted from stochastic simulations. The cellular SOS1 population is fixed at 4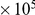 molecules per cell. As in (A), the solid, dashed and dotted lines correspond to fixed bivalent LAT populations (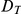) of 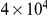, 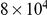 and 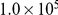 molecules per cell, and Grb2 and monovalent LAT cellular populations are fixed at the same values as in (A). The vertical lines correspond to the theoretically predicted boundaries between the one phase (sol) and two-phase (sol-gel) regions obtained by varying 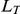 along the horizontal line in (A) corresponding to a fixed 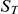 value of 4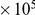 molecules per cell. (**C**) Plot of simulated 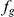 values as a function of 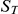 keeping 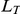 fixed at 1.5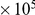 molecules per cell, and Grb2 and monovalent LAT fixed at the same values as in (A). The vertical lines correspond to the theoretically predicted boundaries between the one phase (sol) and two-phase (sol-gel) regions obtained by varying 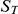 along the vertical line in (A) corresponding to a fixed 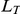 value of 1.5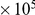 molecules per cell.

We also examine the sol-gel region in the concentration space spanned by the total SOS1 and trivalent LAT concentrations as we previously did in Nag et al. (2009) for the homogeneous case. In Fig. 5 we fix the total Grb2 and monovalent LAT concentrations at 7.5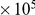 and 4.2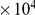 per cell respectively and consider three cases corresponding to three fixed 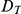 values in the range 4.0
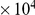
–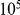 per cell. The boundaries of the one phase (sol) and two-phase (sol-gel) regions for these three cases, Fig. 5a, were obtained using the equilibrium theory. As expected since bivalent LAT blocks branching, the higher is the fixed 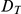 value, the smaller is the two-phase region.

In Fig. 5b, values of 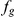 obtained from simulations are plotted as a function of 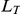, keeping 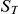, 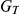 and 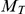 fixed at 4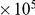, 7.5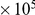 and 4.2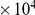 molecules/cell respectively for each of the three fixed 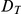 values in Fig. 5a. In Fig. 5c, the 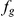 values are plotted as a function of 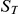 keeping 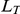 fixed at 1.5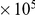 and 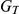, 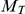 and 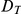 at the same values as in Fig. 5b. The horizontal lines indicate the theoretically predicted boundaries between the one phase and two-phase regions for the different 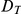 values in Fig. 5a. As in Figs. 4b and 4c, lack of consideration of finite size effects in the theoretical approach results in discrepancies between the theoretically predicted and simulated 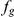 values at the above-mentioned boundaries.

**Figure 6:**
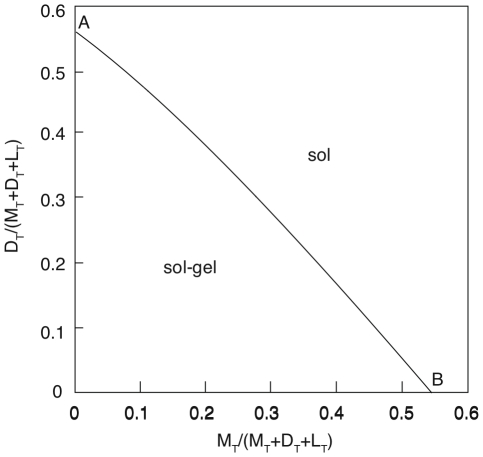
Phase diagram in the concentration space spanned by bivalent and monovalent LAT concentrations. The total LAT (monovalent+bivalent+trivalent) concentration is fixed at 3.45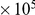 molecules/cell and the Grb2 and SOS1 concentrations are 7.5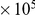 molecules/cell and 3.75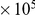 molecules/cell respectively.

To get a better feeling for how sensitive LAT superaggregate formation is to the presence of monovalent and bivalent LAT, we consider in Fig. 6, a fixed population of phosphorylated LAT (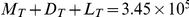 per cell) and show the set of 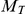, 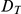 and 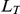 values for which a superaggregate can form. The dotted line in Fig. 6 is the boundary between the one-phase (sol) and the two-phase (sol gel coexistence) regions in the concentration space spanned by monovalent and bivalent LAT. The origin (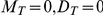, 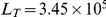 per cell) is in the sol-gel coexistence region. In the absence of monovalent and bivalent LAT, for the values of Grb2 and SOS1 used in Fig. 6, a sol-gel coexistence region is predicted to exist for 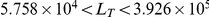. For a mixture of only monovalent and trivalent LAT (the x-axis), when the monovalent LAT is greater than 54% of the total phosphorylated LAT (point A in Fig. 6), corresponding to 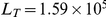 per cell, supperaggregate formation is blocked. Similarly, for a mixture of only bivalent and trivalent LAT (the y-axis), superaggregate formation is blocked when the bivalent LAT concentration is greater than 56% of the total LAT (point B in Fig. 6) corresponding to 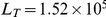 per cell. Monovalent LAT, which terminates aggregate formation, is only slightly more effective in inhibiting superaggregate formation than bivalent LAT, which blocks branching within aggregates, but not the propagation of aggregation. The results shown in Fig. 6 indicate that superaggregate formation can occur in the presence of substantial heterogeneity in the valence of LAT. For this example, superaggregate formation occurs when the sum of the concentrations of monovalent and bivalent LAT is less than 54%.

## Discussion

In T cells and mast cells the scaffolding protein LAT links receptors to downstream signaling events [8], [29]. LAT has three terminal tyrosines, each of which when phosphorylated becomes a binding site for the adaptor protein Grb2, which through its SH2 domain, binds to phosphorylated LAT and through its two SH3 domains binds to the proline-rich regions of SOS1 [6]. Two Grb2 molecules can bind to SOS1 to form a Grb2-SOS1-Grb2 dimer, which can bridge two tyrosine phosphorylated LAT molecules. LAT is phosphorylated by Syk family kinases that have been recruited to the cytoplasmic domains of receptors containing phosphorylated immunoreceptor tyrosine-based activation motifs (ITAMs). Thus, extracellular stimuli, by controlling the level of activation of Syk family kinases can modulate the valence of LAT for Grb2 from zero to three. Large aggregates of LAT have been observed in T cells following T cell stimulation with anti-CD3 antibodies [6], [26] and in mast cells following the aggregation of IgE-Fc
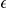
RI complexes [27], [28].

Previously we showed that in the absence of monovalent and bivalent LAT, for a certain range of concentrations of Grb2, SOS1 and trivalent LAT, an equilibrium theory of aggregate formation predicted the Grb2-SOS1-Grb2 mediated formation of a LAT superaggregate [30]. The range of concentrations over which superaggregate formation was predicted to occur corresponded to a sol-gel coexistence region where the sol phase consisted of monomers and small aggregates of LAT, and the gel phase consisted of a single superaggregate of LAT.

In this paper we have presented an equilibrium theory for Grb2-SOS1-Grb2 mediated LAT oligomerization when the LAT population is heterogeneous with respect to its valence for Grb2, extending the equilibrium theory for Grb2-SOS1-Grb2 mediated oligomerization of LAT molecues which are homogeneous with respect to their valence for Grb2 [30]. We have adapted a kinetic Monte Carlo method [32], [34] used in our previous studies [30] to simulate the model and assess the importance of finite size effects. These effects were small and the simulations were in good agreement with predictions of the equilibrium theory (see Fig. 3). In the absence of experimentally derived ratios of mono-, bi- and trivalent LAT, we made *ad hoc* but biologically reasonable assumptions about the relative proportions of the different valence states of LAT. We intend to provide an estimate for the relative levels, in stimulated mast cells, of the various LAT valence states in a forthcoming publication (Monine et al. unpublished), using a computational model of Fc
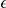
RI-mediated early signaling events in mast cells that would include LAT phosphorylation by activated Syk kinase and LAT dephosphorylation by a pool of phosphatases.

The question that most concerned us was, when we include mono- and bivalent LAT in the presence of trivalent LAT, do we still predict there to be a wide range of concentrations for which superaggregate formation can occur? Monovalent LAT terminates the growth step in aggregate formation while bivalent LAT, although it allows for chain elongation, blocks new branch formation. As expected, in all the cases we considered, the presence of monovalent and bivalent LAT reduced the size of the sol-gel coexistence region. In Fig. 4a, for concentrations of Grb2 and SOS1 found in Jurkat T cells, we have mapped the predicted boundaries of the coexistence region in the space spanned by the bivalent and trivalent LAT concentrations for a set of monovalent LAT concentrations. As can be seen, adding monovalent and bivalent LAT reduces the size of the coexistence region but there is still a substantial range of concentrations where a superaggregate (Fig. 4a) can form that contain significant fractions of the total LAT (Fig. 4b and c). In Fig. 5a we mapped the predicted boundaries of the sol-gel coexistence region in the space of SOS1 and trivalent LAT concentrations, for a set of fixed bivalent LAT concentrations and a single monovalent LAT concentration. Again, although the size of the coexistence region is reduced as the concentration of bivalent LAT is increased, there is still a wide range of concentrations within the boundary of the sol-gel region.

During the initial phase of signaling we expect the distribution of the LAT population over the different valence states to vary with the strength and duration of the external stimulus. In Fig. 6 we consider a fixed concentration of phosphorylated LAT, 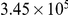 per cell, and Grb2 and SOS1 concentrations typical of a Jurkat T cell. The value of LAT was chosen so that when all the LAT is trivalent, a substantial fraction of it is predicted to be in a superaggregate. When there is no bivalent LAT present, and we increase monovalent LAT while reducing trivalent LAT, we continue to get gel formation until the concentration of monovalent LAT exceeds 54% of the total. When there is no monovalent LAT present, and we increase bivalent LAT while reducing trivalent LAT, gel formation is completely inhibited when the bivalent LAT exceeds 56% of the total. Surprisingly, monovalent LAT is only slightly more effective than bivalent LAT in reducing the sol-gel coexistence region. In order for superaggregate formation to occur there must be a substantial concentration of trivalent LAT. Although the presence of monovalent and bivalent LAT reduces the sol-gel coexistence region, the set of concentrations of Grb2, SOS1, mono-, bi- and trivalent LAT for which superaggregate formation is predicted remains large and includes concentrations we expect occur in activated Jurkat T cells.

Sol-gel coexistence may have been observed in stimulated RBL-2H3 cells. Using immunogold labeling of mast cell membrane sheets in conjunction with transmission electron microscopy, Wilson et al. (2001a) determined the distribution of LAT clusters formed by cross-linking of Fc
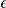
RI-bound IgE by an added multivalent antigen (see Fig. 8 in [27]). A minute after the stimulation, a distribution of LAT aggregate sizes was observed with one aggregate much larger than the rest. The outlying aggregate contained over 160 LAT molecules at two minutes after stimulation. This was a lower bound on the number of LAT molecules in the aggregate since the efficiency of labeling was not determined. RBL-2H3 cells have been estimated to contain 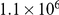 LAT molecules, although it is not known if all of these are on the plasma membrane. The equilibrium theory predicts that in the sol-gel coexistence region a significant fraction of the total LAT should be in a superaggregate. If the entire cell surface was a single well mixed system, we would therefore expect to see hundreds of thousands rather than hundreds of LAT molecules in a single aggregate. One way for our predictions to be consistent with the observations of Wilson et al. [27] would be if the size of the region where LAT can be treated as well mixed is about a thousand times smaller than the surface area of the cell. The concept of a cell surface being made up of many microdomains, is consistent with single particle tracking experiments on RBL-2H3 cells that show diffusing receptors confined for many seconds in regions with a characteristic length scale of microns [39].

In addition to restrictions imposed by cell surface domains, active mechanisms reduce aggregate size. The aggregation of LAT is crucial for the propagation of downstream signaling [6], but as signaling propagates, active mechanisms arise to attenuate signaling. On T cells, shortly after receptor phosphorylation and LAT aggregation, LAT is internalized through a mechanism involving the ubiquitin ligase c-Cbl [40]. LAT internalization requires the presence of c-Cbl, although not the ubiquitylation of LAT, while LAT degradation requires LAT ubiquitylation [41]. Like SOS1, c-Cbl can bind to Grb2 SH3 domains through its proline-rich domains and form Grb2-Cbl-Grb2 complexes that can be incorporated into LAT aggregates [6]. If c-Cbl is in low concentration compared to SOS1, c-Cbl will be preferentially incorporated into large aggregates compared to small aggregates. If two LAT molecules in an aggregate bound to a Grb2-Cbl-Grb2 complex can mediate the internalization of an entire aggregate, c-Cbl will efficiently reduce the concentration of surface LAT and limit the growth of large aggregates. Incorporation of LAT internalization reactions into our existing model would serve to lower the size of the supperaggregate and might result in better agreement between the sizes of the theoretically predicted and experimentally observed superaggregates.

A major assumption of our current model is that we can ignore binding reactions that lead to the formation of closed structures (loops). Assuming we allow loop formation in our model, there would be a number of conceivable ways to form closed structures. One possible way loops can form would involve binding of the two unbound SH2 domains of the Grb2-SOS1-Grb2 cross-linker to two different phosphotyrosine groups on the same LAT molecule. Such binding events which are feasible for only trivalent LAT and bivalent LAT molecules would convert them to monovalent and zero-valent LAT species respectively. Thus LAT molecules that might participate in aggregate formation by increasing a chain length or by seeding a branch would be converted to species which would either participate in chain termination or not participate in aggregate formation at all. Another way of formation of closed structures is the formation of cross-links between two LAT molecules which are already cross-linked. Formation of such loops would involve the consumption of Grb2-SOS1-Grb2 cross-linkers and unbound LAT phosphotyrosines which could otherwise be instrumental in enhancing the aggregate size. An additional way by which loops can form is the cross-linking of the two ends of a linear of branched chain of LAT molecules which would also result in the non-productive use of cross-linkers and LAT phosphotyrosines so far as increasing the aggregate size is concerned. Hence, it is expected that the addition of loop formation reactions to our existing equilibrium model will limit the size of the superaggregate and reduce the discrepancy between the theoretically predicted and experimentally observed superaggregate sizes. Although we could incorporate certain specific types of loops into the model, experiments to date offer little guidance as to what types of loops are most likely to occur. We expect very large loops to be unlikely because of the multiple ways in which they can open and because the probability of a chain closing to form a loop decreases with increasing chain length. As we have pointed out, we don't expect small loops to have a high probability of forming because if they did, we would not expect to see the observed formation of large aggregates of LAT [27]. This leaves us with a large poorly defined set of possible loop closures to consider. We feel it is premature to try to add loops to the model until there is data to show that such structures do form during the aggregation of LAT.

The only adaptor molecule in our model that can form a dimer via binding to SOS1 is Grb2. However, at least another adaptor Grap (Grb-2-like accessory protein) is expressed in T cells, which like Grb2 is composed of one central SH2 domain flanked by two SH3 domains [8], [42], [43]. Grap not only binds the same sites on LAT as GRB2 through its central SH2 domain but also the proline-rich regions of SOS1 through at least one of its two SH3 domains [42], [43] and is hence capable of forming a 2∶1 complex with SOS1, although no experimental evidence to this effect has been reported. A third member of the Grb2 family of adaptor proteins, Gads, binds phosphorylated LAT tyrosines 171 and 191 which are also binding sites for Grb2 [12], [44]. However expermental investigations [6] indicate that Gads does not appear to bind to SOS1 [6], [45] and may act as a monovalent inhibitor of gel formation, reducing the range of concentrations over which superaggregate formation can occur [30].

LAT, LAX and LAB form a family of transmembrane adaptor proteins that are widely expressed in lymphocytes [7]. LAB's three distal tyrosines are its main sites of phosphorylation and all are sites for Grb2 binding [46]. LAX also binds Grb2, and five of its ten tyrosines are in Grb2 -binding motifs [47]. We have confined our discussion to aggregation of LAT by Grb2-SOS1-Grb2 dimers since LAT is the most studied of these three transmembrane adaptors, but LAB and LAX are possible candidates for superaggregate formation as well. Aggregation is ubiquitous in cell signaling, manifesting itself at many levels. Once signaling is initiated, mechanism that result in the formation of large aggregates compete with opposing mechanisms that limit the growth of aggregates. We expect that models of aggregation, tailored to specific signaling complexes, can contribute to our understanding of the multiple roles of aggregation in regulating cell signaling cascades.

## Supporting Information

Appendix S1 **Calculation of dimensionless partition function for LAT population with distribution of valence.**
(PDF)

Appendix S2 **Condition for gel formation for LAT population with mixed valence.**
(PDF)

Appendix S3 **Calculation of dimensionless partition function for a population of bivalent receptors that can be cross-linked by ligand species of variable valency.**
(PDF)
