# Supplementary material for: Modeling and Simulation of Aggregation of Membrane Protein LAT with Molecular Variability in the Number of Binding Sites for Cytosolic Grb2-SOS1-Grb2
Source: PLoS One. 2012 Mar 1;7(3):e28758. doi: 10.1371/journal.pone.0028758 (PMC3291652; doi:10.1371/journal.pone.0028758)
Supplement: Appendix S1 — Calculation of dimensionless partition function for LAT population with distribution of valence. (PDF) [file pone.0028758.s001.pdf]

Appendix S1. Calculation of dimensionless partition function for LAT population with distribution of valence

The n-th equation in this appendix is numbered as (S1-n). For example, the first equation is numbered as (S1-1). All the other equations mentioned in the following derivations refer to equations in the main body of the article.

To include the participation of monovalent LAT in LAT aggregation, the factor $(1+\beta)^n$ in our previous paper [1] has to be replaced by $(1+\beta+\Omega)^n$. The Ω term is included to take into account the possible binding of a monovalent LAT to a binding site on a Grb2 molecule, which is part of a Grb2-SOS1-Grb2 complex, with the other Grb2 in the complex being bound to another LAT molecule with valency greater than one. Incorporating the bivalent LAT species into the aggregates is more tricky. We make the ansatz that in order to include bivalent LAT in the cross-linking species, one should replace in the expression of q, the dimensionless concentration of the Grb2-SOS1-Grb2 species ($c = 2K_{GL}C$) by c', where c' is the sum of c and the dimensionless concentration of all linear chains of bivalent LAT molecules bridged by the Grb2-SOS1-Grb2 complex and having Grb2-SOS1-Grb2 moieties at either end. The sum of the concentrations of all such chains can be written as
$(2K_{GL}C)^2D + (2K_{GL}C)^3(2\overline{K}_{GL}D)D + (2K_{GL}C)^4(2\overline{K}_{GL}D)^2D + ...$
$= D(2K_{GL}C)^2/(1 - 2K_{GL}C2\overline{K}_{GL}D)$. Hence, we can write c' as

$$c' = c + \frac{2\overline{K}_{GL}D(2K_{GL}C)^2}{1-(2K_{GL}C)(2\overline{K}_{GL}D)}. \tag{S1-1}$$

Replacing $2K_{GL}C$ by c from Eq. (8) and $2\overline{K}_{GL}D$ by ϵd (Eqs. (4) and (6)), the above equation can be re-written as

$$c' = c + \frac{\epsilon d c^2}{1-c\epsilon d} = \frac{c}{1-cd\epsilon}. \tag{S1-2}$$

In terms of c', we define

$$q_0' = \frac{\frac{3}{2}\delta' w^2}{1-2\delta' w}, \tag{S1-3}$$

where

$$\delta' = \frac{\alpha c'}{(1+\beta+\Omega)^2}, \tag{S1-4}$$

and

$$w = l(1+\beta+\Omega)^3. \tag{S1-5}$$

Following the procedure in Goldstein et al. [2], it can be shown that the second term on the RHS of Eq. (14) can be written as

$$q_{(2)} = 4q_0 \sum_{m=0}^{m=\infty} \frac{(1/2)...(1/2-m)}{(m+1)!}(-4q_0^2)^m \frac{\gamma^m}{(m+2)}, \tag{S1-6}$$

where

$$\gamma = \frac{1}{(3w/2)^2}. \tag{S1-7}$$

By virtue of its definition, the term q_0' represents the dimensionless partition function for all linear chains composed of bivalent and trivalent LAT molecules in which the terminal LAT molecules are trivalent. Replacing q_0 by q_0' in Eq. (S1-6) will account for all LAT aggregates with only trivalent LAT molecules in the terminal positions. We observe that we can include the concentrations of LAT aggregates, which have

one or more bivalent terminal LAT molecule(s) at terminal positions, by multiplying the term containing γ^m on the RHS of Eq. (S1-6) by η^{m+2}, where

$$\eta = \frac{\alpha l(1+\beta+\Omega)^2 + \epsilon d(1+\beta+\Omega)}{\alpha l(1+\beta+\Omega)^2} = 1 + \frac{\epsilon d}{\alpha l(1+\beta+\Omega)}. \tag{S1-8}$$

Thus, to obtain the dimensionless partition function for a heterogeneous LAT population with variable valency, the second term on the RHS of Eq. (14) should be replaced by

$$q'_{(2)} = 4q'_0 \sum_{m=0}^{m=\infty} \frac{(1/2)...(1/2-m)}{(m+1)!}(-4{q'_0}^2)^m \frac{\gamma^m \eta^{m+2}}{(m+2)}. \tag{S1-9}$$

We define

$$\gamma' = \frac{\gamma}{\eta^3}, \tag{S1-10}$$

and

$$q_v = q'_0 \eta^2. \tag{S1-11}$$

In terms of these newly defined quantities, we can write

$$q'_{(2)} = 4q_v \sum_{m=0}^{m=\infty} \frac{(1/2)...(1/2-m)}{(m+1)!}(-4q_v^2)^m \frac{(\gamma')^m}{(m+2)}. \tag{S1-12}$$

On comparing Eqs. (S1-12) and (S1-6), we observe that the above equation can be written as

$$q'_{(2)} = \frac{1}{\gamma' q_v}\left[1 - \frac{1-(1-4\gamma' q_v^2)^{\frac{3}{2}}}{6\gamma' q_v^2}\right]. \tag{S1-13}$$

To obtain the complete partition function we have to add the reduced concentrations of three types of species :
i) species containing only monovalent LAT molecule(s) and up to two such molecules (the contribution to the complete partition function of such species is $(M_T/L_T)m(1+\beta+\Omega/2)$),
ii) species containing a single bivalent LAT molecule, and capped by up to two monovalent LAT molecules (the contribution to the total partition function of such species is $(D_T/L_T)d(1+\beta+\Omega)^2$),
and,
iii) species containing a single trivalent LAT molecule and capped by up to three monovalent LAT molecules (the contribution to the total partition function of such species is $l(1+\beta+\Omega)^3$).
Thus, we obtain the total partition function q for LAT molecules with variable valency as

$$q = \frac{M_T}{L_T}m(1+\beta+\Omega/2) + \frac{D_T}{L_T}d(1+\beta+\Omega)^2 + l(1+\beta+\Omega)^3 + \frac{1}{\gamma' q_v}\left[1 - \frac{1-(1-4\gamma' q_v^2)^{\frac{3}{2}}}{6\gamma' q_v^2}\right]. \tag{S1-14}$$

The terms M_T/L_T and D_T/L_T appear in the above equation because we normalize all the concentration terms by the total trivalent LAT concentration L_T.

References

1. Nag A, Monine M, Faeder JR, Goldstein B (2009) Aggregation of membrane proteins by cytosloic cross-linkers: Theory and simulation of the LAT-Grb2-SOS1 system. Biophys J 96: 2604-2623.

2. Goldstein B, Perelson AS (1984) Equilibrium theory for the clustering of bivalent cell surface receptors by trivalent ligands: Application to histamine release from basophils. Biophys J 45: 1109-1123.
