# Supplementary material for: Modeling and Simulation of Aggregation of Membrane Protein LAT with Molecular Variability in the Number of Binding Sites for Cytosolic Grb2-SOS1-Grb2
Source: PLoS One. 2012 Mar 1;7(3):e28758. doi: 10.1371/journal.pone.0028758 (PMC3291652; doi:10.1371/journal.pone.0028758)
Supplement: Appendix S2 — Condition for gel formation for LAT population with mixed valence. (PDF) [file pone.0028758.s002.pdf]

Appendix S2. Condition for gel formation for LAT population with mixed valence

The n-th equation in this appendix is numbered as (S2-n). For example, the first equation is numbered as (S2-1). An equation numbered as (S1-n) refers to the n-th equation of Appendix S1. All the other equations mentioned in the following derivations refer to equations in the main body of the article.

Using Eqs. (19) to (22), Eq. (18) can be re-written as

$$q = \frac{M_T}{L_T} m(1+\beta+\Omega/2) + \frac{D_T}{L_T} d(1+\beta+\Omega)^2 + w\left[1+3\eta z - 2z^3 + 2(z^2-\eta)^{\frac{3}{2}}\right], \tag{S2-1}$$

where w is given by Eq. (S1-5), η by Eq. (S1-8) and

$$z = \frac{1-2\delta' w}{2\delta' w}. \tag{S2-2}$$

Using the definitions of M_1 and q in Eqs. (36) and (S2-1) respectively, we can show that

$$\begin{aligned} M_1 &= l\left(\frac{\partial q}{\partial l}\right) + d\left(\frac{\partial q}{\partial d}\right) + m\left(\frac{\partial q}{\partial m}\right) \\ &= \left(\frac{M_T}{L_T}\right) m(1+\beta+\Omega) + \left(\frac{D_T}{L_T}\right) d(1+\beta+\Omega)^2\left(1+\frac{2\Omega}{1+\beta+\Omega}\right) \\ &+ w\left(1+\frac{3\Omega}{1+\beta+\Omega}\right)\left[1+3\eta z-2z^3+2(z^2-\eta)^{\frac{3}{2}}\right] \\ &- w\frac{(\eta-1)\Omega}{1+\beta+\Omega}\left[3z-3(z^2-\eta)^{\frac{1}{2}}\right] \\ &- w(z+1)\left(\frac{1}{1-cd\epsilon}+\frac{\Omega}{1+\beta+\Omega}\right)\left[3\eta-6z^2+6z(z^2-\eta)^{\frac{1}{2}}\right]. \end{aligned} \tag{S2-3}$$

According to Eq. (37), M_2 can be expressed as

$$M_2 = l\left(\frac{\partial M_1}{\partial l}\right) + d\left(\frac{\partial M_1}{\partial d}\right) + m\left(\frac{\partial M_1}{\partial m}\right). \tag{S2-4}$$

From Eqs. (S2-3) and (S2-4), it can be shown that

$$M_2 = M_{21}+M_{22}+M_{23}+M_{24}+M_{25}+M_{26}+M_{27}+M_{28}, \tag{S2-5}$$

where

$$M_{21} = \left(\frac{M_T}{L_T}\right) m(1+\beta+\Omega)\left(1+\frac{\Omega}{1+\beta+\Omega}\right), \tag{S2-6}$$

$$M_{22} = \left(\frac{D_T}{L_T}\right) d(1+\beta+\Omega)^2\left[1+\frac{6\Omega}{1+\beta+\Omega}+2\left(\frac{\Omega}{1+\beta+\Omega}\right)^2\right], \tag{S2-7}$$

$$M_{23} = w\left[1+3\eta z-2z^3+2(z^2-\eta)^{\frac{3}{2}}\right]\left[1+\frac{9\Omega}{1+\beta+\Omega}+6\left(\frac{\Omega}{1+\beta+\Omega}\right)^2\right], \tag{S2-8}$$

$$M_{24} = -\frac{w(\eta-1)\Omega}{1+\beta+\Omega}\left[3z-3(z^2-\eta)^{\frac{1}{2}}\right]\left(3+\frac{4\Omega}{1+\beta+\Omega}\right), \tag{S2-9}$$

$$\begin{aligned} M_{25} &= -\left[3\eta-6z^2+6z(z^2-\eta)^{\frac{1}{2}}\right]\left[\frac{(1+\beta+\Omega)^2}{2\alpha c}+\frac{2\Omega(1+\beta+\Omega)}{\alpha c}\right. \\ &\quad \left. +w(z+1)\left(\frac{3\Omega}{1+\beta+\Omega}\right)+w(z+1)\left(\frac{2\Omega}{1+\beta+\Omega}\right)^2\right], \end{aligned} \tag{S2-10}$$

$$M_{26} = \frac{(1+\beta+\Omega)(1+\beta+2\Omega-cd\epsilon\Omega)}{2\alpha c}\frac{3(\eta-1)\Omega}{1+\beta+\Omega}, \tag{S2-11}$$

$$\begin{aligned} M_{27} \quad = \quad & -\tfrac{(1+\beta+\Omega)(1+\beta+2\Omega-cd\epsilon\Omega)}{2\alpha c}\left[\tfrac{(1+\beta+\Omega)^2}{\alpha c w}\left\{6z - 3(z^2-\eta)^{\frac{1}{2}}\right\}\right. \\ & \left. +\tfrac{\Omega}{1+\beta+\Omega}\left\{3(1-\eta)+12z(z+1)-6(z+1)(z^2-\eta)^{\frac{1}{2}}\right\}\right], \end{aligned} \tag{S2-12}$$

and

$$M_{28} = \frac{3(1+\beta+\Omega)}{2\alpha c w(z^2-\eta)^{\frac{1}{2}}}\left[(1+\beta+2\Omega) - \left(\frac{1-cd\epsilon}{2\alpha c l}\right)\left\{1+\frac{\Omega}{1+\beta+\Omega}(1-cd\epsilon)\right\}\right]. \tag{S2-13}$$

From Eqs. (S2-5)-(S2-13), we find by observation that for $z^2 = \eta$, $M_2 \to \infty$. Thus, at the gel point,

$$(z+\eta^{\frac{1}{2}})(z-\eta^{\frac{1}{2}}) = 0. \tag{S2-14}$$

The above equation in z has two roots, of which only the positive root $z = \eta^{\frac{1}{2}}$ is physically acceptable, so that the following equation in z holds at the gel point.

$$z = \eta^{\frac{1}{2}} \tag{S2-15}$$

Using Eqs. (20), (23), (S1-5) and (S2-2) for η, δ', w and z respectively, the above equation can be re-written as

$$\frac{1-cd\epsilon}{2\alpha c l(1+\beta+\Omega)} = 1 + \left[1+\frac{\epsilon d}{\alpha l(1+\beta+\Omega)}\right]^{\frac{1}{2}}. \tag{S2-16}$$
