# Supplementary material for: Modeling and Simulation of Aggregation of Membrane Protein LAT with Molecular Variability in the Number of Binding Sites for Cytosolic Grb2-SOS1-Grb2
Source: PLoS One. 2012 Mar 1;7(3):e28758. doi: 10.1371/journal.pone.0028758 (PMC3291652; doi:10.1371/journal.pone.0028758)
Supplement: Appendix S3 — Calculation of dimensionless partition function for a population of bivalent receptors that can be cross-linked by ligand species of variable valency. (PDF) [file pone.0028758.s003.pdf]

Appendix S3. Calculation of dimensionless partition function for a population of bivalent receptors that can be cross-linked by ligand species of variable valency

First we review the results obtained by Goldstein et al. [1] on the clustering of bivalent cell surface receptors by trivalent ligands. The total non-dimensional partition function which is the sum of all receptor containing species, normalized by the total receptor concentration X_T is given by

$$q = \frac{1}{\gamma q_0}\left[1 - \frac{1-(1-4\gamma q_0^2)^{3/2}}{6\gamma q_0^2}\right], \tag{S3-1}$$

where q_0 is the reduced partition function for all linear chains of bivalent receptors linked by trivalent ligands. It can be shown that

$$q_0 = w/(1-\delta w), \tag{S3-2}$$

where

$$w = (1+\chi)^2 x, \tag{S3-3}$$

given that $x = X/X_T$ and $\chi = 3KC$, where X and C are respectively the equilibrium concentrations of free receptors and ligands, and K is the equilibrium constant for the binding of a single site on a ligand in solution to a single cell surface receptor site.

$$\delta = \alpha\chi/(1+\chi)^2, \tag{S3-4}$$

where

$$\alpha = 4K_x X_T, \tag{S3-5}$$

and K_x is the first cross-linking constant.

$$\gamma = \frac{\chi\alpha\beta}{(1+\chi)^3}, \tag{S3-6}$$

where

$$\beta = K_{xx} X_T, \tag{S3-7}$$

and K_{xx} is the second cross-linking constant, which describes the binding to a single receptor site of a site on a ligand that already has two sites bound to two other receptors.

In order to generalize the theory for bivalent cell surface receptor and trivalent ligand to one for bivalent receptor and a mixed population of monovalent, bivalent and trivalent ligands, we have to modify q_0. Now q_0 in Eq. (S3-1) can be represented as $q_0 = q_f(1+\chi)^2$ and q_0 can be systematically modified by modifying each of its factors. We first modify q_f which is the dimensionless partition function for linear chains which both begin and end with a free receptor site.

Let the equilibrium concentration of free bivalent ligand and monovalent ligand be represented by D and M, respectively, and let the corresponding total concentrations be D_T and M_T respectively. We assume that the equilibrium constant K is the same for ligands with different valencies. For convenience, we define the following dimensionless quantities.

$$\xi = 3KC_T, \kappa = KD_T, \zeta = KM_T, \tag{S3-8}$$

and,

$$c = C/C_T, d = D/D_T, m = M/M_T. \tag{S3-9}$$

Hence, we can write

$$\chi = \xi c. \tag{S3-10}$$

We also define Δ and μ as

$$\Delta = KD = \kappa d; \mu = KM = \zeta m. \tag{S3-11}$$

We can divide all linear chains beginning and ending with a free receptor into two categories
i) chains which are cross-linked only by bivalent ligands (includes the free receptor), and
ii) chains which are cross-linked by both bivalent and trivalent receptors.
The partition function corresponding to category (i) can be expressed as

$$q_f^{(a)} = \left[X + (4KK_xDX) + (4KK_xDX)^2 + ...\right]/X_T = x/(1-\alpha\Delta x). \tag{S3-12}$$

The partition function for category (ii) can be obtained starting from the partition function of all linear chains which are cross-linked only by trivalent ligands and have at least two receptors, since the free receptor has already been included in category (i). The partition function $q_f^{(3)}$ of linear chains with at least two receptors, containing only trivalent ligands and having a free receptor at both ends can be obtained by subtracting the contribution of the free receptor from q_f in Goldstein et al. [1]. Hence,

$$q_f^{(3)} = \frac{x}{1-\alpha\chi x} - x = \frac{\alpha\chi x^2}{1-\alpha\chi x}. \tag{S3-13}$$

The partition function for category (ii), which we call $q_f^{(b)}$, can easily be obtained by substituting x by $q_f^{(a)}$ in the final form of $q_f^{(3)}$. Thus the final expression for q_f is

$$q_f = q_f^{(a)} + q_f^{(b)} = q_f^{(a)} + \frac{\alpha\chi(q_f^{(a)})^2}{1-\alpha\chi(q_f^{(a)})} = (\frac{x}{1-\alpha\Delta x})/(1-\frac{\alpha\chi x}{1-\alpha\Delta x}). \tag{S3-14}$$

We can also obtain the same expression for q_f starting from a sequence generating function approach. In this approach, we consider two different sequences,
i) sequence of all linear chains, cross-linked only by trivalent ligands, containing at least two receptors, and having free receptor sites at both ends, and
ii) sequence of all linear chains, starting from the free receptor, cross-linked only by bivalent ligands and having free receptor sites at both ends.

Sequence (i) is represented as $U = \{u_2, u_3, u_4...\}$ and sequence (ii) as $V = \{v_1, v_2, v_3, v_4, ...\}$. Thus the element in each sequence with subscript k denotes the linear chain in that sequence containing k receptors. Note that the U sequence does not have any u_1 because the free receptor is included in the Vsequence. It can be shown that all the linear chains which are cross-linked by both bivalent and trivalent ligands can be generated using the elements of the U and V sequences, where we consistently use the bivalent ligand as the linker between the elements of the U and V sequences. We denote the dimensionless sum of concentrations of all the elements in the U sequence by S_U and the sum of concentrations of all the elements in the V sequence by S_V. The sums are rendered dimensionless on division by the total number of receptors X_T.

Consider the chains which starts with an U element and ends with a V element. We term this set as the UV set. If we add another U element to the chains in this UV set we get the UVU set. Next consider the following sets which are the subsets of the set of all chains which are cross-linked by both bivalent and trivalent ligands -
i) $UV + UVUV + UVUVUV + ...$
ii) $UVU + UVUVU + UVUVUVU + ...$
iii) $VUV + VUVUV + VUVUVUV + ...$
The partition functions for sets (i)-(iii) can be evaluated as

$$q_{uv}^{(i)} = \left[4(2KK_xDX_T)S_US_V\right]/\left[1-(2KK_xDX_T)^2(4S_US_V)\right], \tag{S3-15}$$

$$q_{uv}^{(ii)} = \left[4(2KK_xDX_T)^2 S_U^2 S_V\right] / \left[1 - (2KK_xDX_T)^2(4S_US_V)\right], \tag{S3-16}$$

and

$$q_{uv}^{(iii)} = \left[4(2KK_xDX_T)^2 S_U S_V^2\right] / \left[1 - (2KK_xDX_T)^2(4S_US_V)\right]. \tag{S3-17}$$

In order to obtain the total partition function for all linear chains in which receptors are cross-linked by both bivalent and trivalent ligands, we should consider the set of elements of the U sequence, connected only by bivalent ligands. Such elements comprise the set $\{UU, UUU, UUUU, ...\}$ and the sum of the concentrations of the elements of this sequence is denoted by S_U. Moreover, we have to incorporate elements of this sequence into the chains in each of sets (i)-(iii). It can be shown that on incorporating the elements of this set into the sets (i), (ii) and (iii), the corresponding partition functions $q_{uv}^{(i)}$, $q_{uv}^{(ii)}$ and $q_{uv}^{(ii)}$ are modified to yield

$$q_{uv}^{(i)} = [4(2KK_xDX_T)(S_U + S_{UU})S_V] / \left[1 - (2KK_xDX_T)^2\{4(S_U + S_{UU})S_V\}\right], \tag{S3-18}$$

$$q_{uv}^{(ii)} = \left[4(2KK_xDX_T)^2(S_U + S_{UU})^2 S_V\right] / \left[1 - (2KK_xDX_T)^2\{4(S_U + S_{UU})S_V\}\right], \tag{S3-19}$$

and

$$q_{uv}^{(iii)} = \left[4(2KK_xDX_T)^2(S_U + S_{UU})S_V^2\right] / \left[1 - (2KK_xDX_T)^2\{4(S_U + S_{UU})S_V\}\right]. \tag{S3-20}$$

Then, we can express the partition function of all chains (including the free receptor), which have a free receptor site at either end as

$$q_f = S_U + S_{UU} + S_V + q_{uv}^{(i)} + q_{uv}^{(ii)} + q_{uv}^{(iii)}, \tag{S3-21}$$

which reduces to

$$\begin{aligned} q_f &= (S_U + S_{UU} + S_V) \\ &+ [4(2KK_xDX_T)(S_U + S_{UU})S_V(1 + (2KK_xDX_T)(S_U + S_{UU}) \\ &+ (2KK_xDX_T)S_V)]/[1 - (2KK_xDX_T)^2\{4(S_U + S_{UU})S_V\}]. \end{aligned} \tag{S3-22}$$

Now S_U is the same as $q_f^{(a)}$ in Eq. (S3-12) and S_V is the same as $q_f^{(3)}$ in Eq. (S3-13). We can obtain an expression for S_{UU} in the following fashion. Let us consider the sequence of all linear chains which have a free receptor site at either end, cross-linked only by bivalent ligands and containing at least two receptors. The sum of concentrations of the elements in this sequence is given by

$$S_V - x = \alpha\Delta x^2/(1 - \alpha\Delta x). \tag{S3-23}$$

In order to obtain an expression for S_{UU}, we should replace the reduced receptor concentration x by in the above equation by the dimensionless sum of concentrations of all linear chains comprising of receptors cross-linked only by trivalent receptors. This latter sum is thus the same as S_U. Thus,

$$S_{UU} = \alpha\Delta(\alpha\chi x^2/(1 - \alpha\chi x))^2/(1 - \alpha\Delta(\alpha\chi x^2/(1 - \alpha\chi x))), \tag{S3-24}$$

so that,

$$\begin{aligned} S_U + S_{UU} &= \alpha\chi x^2/(1 - \alpha\chi x) \\ &+ \alpha\Delta(\alpha\chi x^2/(1 - \alpha\chi x))^2/(1 - \alpha\Delta(\alpha\chi x^2/(1 - \alpha\chi x))) \\ &= \alpha\chi x^2/(1 - \alpha\chi x - \alpha^2\chi\Delta x^2). \end{aligned} \tag{S3-25}$$

In terms of $(S_U + S_{UU})$ and S_V, q_f can be re-written as

$$q_f = [(S_U + S_{UU}) + S_V + 2\alpha\Delta(S_U + S_{UU})S_V]/(1 - (\alpha\Delta)^2(S_U + S_{UU})S_V). \tag{S3-26}$$

Substituting the values of $(S_U + S_{UU})$ from Eq. (S3-25), and S_V (which is the same as $q_f^{(3)}$) from Eq. (S3-13) into the above equation, we get

$$q_f = x/(1 - \alpha\chi x - \alpha\Delta x) = (\frac{x}{1 - \alpha\Delta x})/(1 - \frac{\alpha\chi x}{1 - \alpha\Delta x}), \tag{S3-27}$$

which is the same as Eq. (S3-14).

The factor $(1 + \chi)^2$ on the RHS of $q_0 = q_f(1 + \chi)^2$ in Goldstein et al. [1] has to be replaced by $(1 + \chi + 2\Delta + \mu)^2$ to account for the bivalent and monovalent ligands which can occupy the end positions of the chains. Hence, the dimensionless partition function for all linear chains (including the free receptor) is given by

$$q_0 = \left[(\frac{x}{1 - \alpha\Delta x})/(1 - \frac{\alpha\chi x}{1 - \alpha\Delta x})\right](1 + \chi + 2\Delta + \mu)^2. \tag{S3-28}$$

Since it is the ligand and not the receptor that has variable valency, Eq. (S3-1) holds for the q_0 in Eq. (S3-28).

References

1. Goldstein B, Perelson AS (1984) Equilibrium theory for the clustering of bivalent cell surface receptors by trivalent ligands: Application to histamine release from basophils. Biophys J 45: 1109-1123.
